# Visual Acuity in Patients Requiring Intravitreal Injections: Short-Term and Long-Term Effects of Delay in Care

**DOI:** 10.1177/24741264221136637

**Published:** 2022-12-16

**Authors:** Weilin Song, Emese Kanyo, Riley Bastian, Rishi P. Singh, Aleksandra V. Rachitskaya

**Affiliations:** 1 Stein Eye Institute, University of California, Los Angeles, Los Angeles, CA, USA; 2 Cleveland Clinic Lerner College of Medicine, Cleveland, OH, USA; 3 Northeast Ohio Medical University, Rootstown, OH, USA; 4 Center for Ophthalmic Bioinformatics, Cole Eye Institute, Cleveland Clinic Foundation, Cleveland, OH, USA; 5 Cole Eye Institute, Cleveland Clinic Foundation, Cleveland, OH, USA

**Keywords:** anti-VEGF, delays in care, intravitreal injections, pandemic, retina, visual acuity

## Abstract

**Purpose:** To assess the short-term and long-term effects of a delay in care on visual acuity (VA) in patients requiring intravitreal injections. **Methods:** This retrospective cohort study comprised patients with neovascular age-related macular degeneration (nAMD), diabetic macular edema (DME), or retinal vein occlusion (RVO) receiving intravitreal injections. The visual and anatomic outcomes at the next completed visit and at the 1-year follow-up were studied. **Results:** Of 1172 patients, 38% had a delay in care (mean 5.7 weeks). Compared with baseline, these patients lost VA (Early Treatment Diabetic Retinopathy Study letters) (mean −2.13 ± 0.49 SE) in the short-term (*P* = .0003) and had a thicker central subfield. Patients with no delay in care had a net VA gain (0.97 ± 0.39) (*P* = .0067). There was no difference in VA between 1 year and the baseline in either group. Long term, patients with nAMD in both groups had VA loss (no delay in care: −1.76 ± 0.60; delayed care: −2.44 ± 0.78) (*P* = .0005 and *P* = .0114, respectively). Patients with DME and no delay in care maintained gains in vision (4.68 ± 1.86) but those with delayed care did not (1.72 ± 2.24) (*P* = .0202 and *P* = .3756, respectively). In both groups, patients with RVO had no significant difference in vision from baseline. **Conclusions:** In patients requiring intravitreal injections, a delay in care of 5.7 weeks affected vision outcomes in the short term but not the long term.

## Introduction

Intravitreal injections are the mainstay therapy for neovascular age-related macular degeneration (nAMD),^1,2^ diabetic macular edema (DME),^3–5^ and retinal vein occlusion (RVO) with macular edema.^6,7^ Several pivotal clinical trials have shown the effectiveness of frequent antivascular endothelial growth factor (VEGF) injections in improving and maintaining visual acuity (VA) in patients with these diseases. However, the high frequency of these injections imposes a significant treatment burden. Nonadherence with treatment recommendations in clinical settings has been shown to be a significant contributor to poor visual outcomes.^8–10^

Studying the effects of delays in treatment with intravitreal injections can be challenging given the heterogeneous patient population and numerous treatment paradigms. The recent COVID-19 pandemic provided an unprecedented opportunity to examine the results of a delay in care in many medical specialties. Our group previously reported that in patients requiring frequent intravitreal injections, an unintended delay in care by 5.3 weeks resulted in significant short-term vision loss.^11^ Although this was observed in all patients, the difference in vision outcomes was most striking in patients with DME and RVO.

The current study addressed the long-term effects of a delay in care. We wondered whether the vision loss in the early follow-up period persisted at 1 year. We also examined imaging findings and the potential causes of a loss of 3 lines or more of VA over the short term and long term in patients with nAMD, DME, or RVO requiring frequent intravitreal injections.

## Methods

### Study Design

This retrospective cohort study was performed at Cole Eye Institute, Cleveland, OH, USA, after approval from the Cleveland Clinic Institutional Review Board. Because of the retrospective nature of the study, written informed consent was not required. All study-related procedures were performed in accordance with good clinical practice, the Declaration of Helsinki, and the US Health Insurance Portability and Accountability Act.

A comprehensive electronic chart review was performed of all adult patients scheduled to see a retina provider at Cleveland Clinic Cole Eye Institute from March 16, 2020, to May 4, 2020. This period was chosen because on March 14, 2020, a state of emergency was declared as a result of the COVID-19 pandemic and clinic downsizing was implemented. The reactivation of outpatient clinics was initiated on May 4, 2020.

Patients were included in this study if they were 18 years or older; had a diagnosis of DME, nAMD, or RVO; had a scheduled visit from March 16, 2020, to May 4, 2020; had an intravitreal injection in the previous 12 weeks (baseline visit), and had a follow-up visit at least 12 months after the baseline visit. Visits not scheduled with a retina provider were excluded. For patients receiving treatment in both eyes, the most recently treated eye was included in the study.

The first scheduled study visit was categorized as completed or missed (canceled or no-show). For patients who missed that first scheduled visit and thus had a delay in care, the next completed visit was used to assess vision. The last completed visit before April 30, 2021, for each patient with a 12-month follow-up was also documented. Therefore, the VA was assessed at the baseline visit (the last visit before the study COVID-19 period), the next completed visit (the scheduled visit or, if missed, the next completed visit), and the last completed visit. The central subfield thickness (CST) was documented using optical coherence tomography (OCT) (Cirrus High-Definition device, V.9.5.1, Carl Zeiss Meditec, Inc) at each of the 3 visits.

Baseline demographics (age, sex, diagnosis, location of treatment [regional or main campus office], and type of intravitreal injection [anti-VEGF or other]) were collected. At each visit, the VA was measured using a Snellen chart; the values were converted to Early Treatment Diabetic Retinopathy Study (ETDRS) letters for analysis.^12^ Subgroup analysis of patients who had lost 15 or more ETDRS letters was performed, and the cause of vision loss was identified by chart review and separated into the following categories: (1) worsening retinal fluid, (2) neovascular (progression or development of tractional retinal detachment, vitreous hemorrhage, neovascular glaucoma, or macular hemorrhage), and (3) atrophic (progression or development of atrophy, ischemia, fibrosis/scar). For patients who initiated treatment on or before March 13, 2019 (a year before the study period), the total number of injections received during the previous years (March 13, 2019, to March 15, 2020) and the study year (March 16, 2020, to March 19, 2021) was counted.

The primary endpoints were changes in VA from the baseline visit to the next completed visit and last completed visit. Additional endpoints included the mean CST at each visit, change in VA stratified by diagnosis, and causes of significant vision loss (≥15 ETDRS letters).

### Statistical Analysis

Categorical variables were described using frequencies and percentages. Continuous variables were described using means with standard errors or medians with the interquartile range. Relationships between categorical variables were assessed using the Fisher exact test and chi-square test, and relationships between continuous variables were assessed using an independent-samples *t* test, analysis of variance, and matched-pair test for means and Mann-Whitney test for medians. Multivariable linear regression models were used to assess relationships between multiple predictors and a continuous dependent variable (change in VA). The Bonferroni correction was used to adjust for multiplicity, and a significance level of *P* = .025 was used for the primary outcomes. Analyses were performed using JMP Pro 14 software (SAS Institute).

## Results

### Demographics

Of the 1172 patients who met the inclusion criteria, 725 (62%) completed the scheduled visit (completed-care group) while 447 (38%) did not (delayed-care group) (Supplemental Figure 1). Table 1 shows the demographics of the study population. The group that missed the scheduled visit had a significantly greater proportion of women (*P* = .0091). There was no significant difference in age, diagnosis (nAMD, DME, or RVO), location of visit (main campus or satellite), or type of intravitreal injection (anti-VEGF or other) between the 2 groups. For patients who missed the scheduled visit and had delayed care, the interval between the visits increased on average by 5.7 weeks (95% CI, 5.3-6.2).

**Table 1.** Patient Demographics.

Parameter	Completed (n = 725)	Delayed (n = 447)	*P* Value
Age (y)			
Median	77	79	.1135^a^
IQR	68, 84	68, 86	
Female sex, n (%)	412 (57)	289 (65)	.0091^b,c^
Diagnosis, n (%)			
nAMD	469 (65)	280 (63)	.6999^b^
DME	102 (14)	69 (15)	
RVO	154 (21)	98 (22)	
Satellite clinic, n (%)	476 (66)	298 (67)	.7618^b^
Anti-VEGF intravitreal injection, n (%)	707 (98)	438 (98)	.60291^b^
Time from baseline to next completed visit			
Weeks	6.8	12.6	<.0001^a,c^
95% CI	6.6, 7.2	12.3, 12.9	
Time from baseline to last completed visit			
Weeks	54.8	52.9	.0007^a^
95% CI	54.1, 55.5	52.1, 53.8	

Abbreviations: DME, diabetic macular edema; IQR, interquartile range; nAMD, neovascular age-related macular degeneration; RVO, retinal vein occlusion; VEGF, vascular endothelial growth factor.

a Student *t* test.

b Chi-square test.

c Statistically significant.

### Visual Acuity

Supplemental Figure 2 shows the best-corrected VA (BCVA) at each of the 3 visits. Patients who missed the scheduled visit had significantly worse VA (ETDRS letters) at the baseline visit (mean—completed care: 63.9 ± 0.77; delayed care: 61.2 ± 0.97) (*P* = .0309), the next completed visit (completed care: 64.8 ± 0.77; delayed care: 59.1 ± 0.98) (*P* < .0001), and the last completed visit (completed care: 63.2 ± 0.82; delayed care: 59.6 ± 1.04) (*P* = .0072).

Supplemental Table 1 compares the visual outcomes (ETDRS letters) by diagnosis (nAMD, DME, or RVO) between patients who received on-time care and those who had delayed care. Patients who missed the first scheduled visit lost VA, while those who completed the visit had a net gain (−2.13 ± 0.49 vs 0.97 ± 0.39) (*P* < .0001). This was most pronounced in those with DME or RVO. By 1 year, there was no statistically significant difference between patients who completed the first visit and those who missed the first scheduled visit overall (−0.68 ± 0.51 vs −1.57 ± 0.68) (*P* = .3104); this was true after stratification by disease. Even after adjusting for age, sex, and baseline VA, patients with a delay in care lost vision compared with patients who had no delay in care in the short term (delayed care: −1.58 ± 0.26) (*P* < .0001); however, there was no statistically significant difference 1 year later (−0.51 ± 0.42) (*P* = .2207).

Table 2 compares the change in BCVA from baseline to the next completed visit and from baseline to the last completed visit. Although there was no significant difference overall, there was a significant difference within each group.

**Table 2.** Change in VA Between Visits in Patients Who Completed a Scheduled Visit vs Those Who Had Delayed Care.

	Mean Change in ETDRS Visual Acuity ± SE			
Group	From Pre to First Completed Visit	*P* Value^a^	From Pre to Last Completed Visit	*P* Value^a^
Entire cohort				
Overall	−0.28 ± 0.29	.3425	−1.09 ± 0.42	.0093^b^
Completed	0.97 ± 0.39	.0067^b^	−0.68 ± 0.51	.1080
Delayed	−2.13 ± 0.49	.0003^b^	−1.57 ± 0.68	.0373
nAMD				
Overall	0.43 ± 0.31	.1623	−2.01 ± 0.48	<.0001^b^
Completed	0.11 ± 0.39	.7223	−1.76 ± 0.60	.0005^b^
Delayed	−1.34 ± 0.50	.0203^b^	−2.44 ± 0.78	.0114^b^
DME				
Overall	1.63 ± 0.94	.0860	3.02 ± 1.36	.0242^b^
Completed	4.18 ± 1.33	.0021^b^	4.68 ± 1.86	.0202^b^
Delayed	−1.42 ± 1.61	.5568	1.72 ± 2.24	.3756
RVO				
Overall	−1.15 ± 0.82	.1657	−1.17 ± 0.95	.2218
Completed	1.46 ± 1.02	.0872	−0.97 ± 1.22	.4164
Delayed	−5.23 ± 1.28	.0012^b^	−1.46 ± 1.53	.3539

Abbreviations: DME, diabetic macular edema; ETDRS, Early Treatment Diabetic Retinopathy Study; nAMD, neovascular age-related macular degeneration; RVO, retinal vein occlusion.

a Matched-pair test; Bonferroni adjustment performed for multiple comparisons.

b Statistically significant.

A comparison between baseline and the first completed visit found that patients who had delayed care lost VA over that period (*P* = .0003) while patients who completed the scheduled visit had a net gain (*P* = .0067). Patients with nAMD and those with RVO who had delayed care had significant short-term vision loss over that period (*P* = .0203 and *P* = .0012, respectively). Patients with DME and timely care had a significant short-term VA gain (*P* = .0021) (Table 2).

A comparison between baseline and the last completed visit showed no statistically significant difference in VA in the group with a delay in care and in the group with no delay in care. Overall, patients requiring intravitreal injections lost VA. Patients with nAMD in both groups lost VA (completed care: *P* = .0005; delayed care: *P* = .0114), while patients with DME and RVO in the delayed-care group did not have significant differences in VA from baseline (*P* = .3756 and *P* = .3539, respectively). Patients with DME who completed the scheduled visit maintained a net gain in VA at 1 year (*P* = .0202) (Table 2).

### Central Subfield Thickness

Out of 1172 patients, 923 had OCT imaging with CST values at every study visit and were included in the mean CST analysis; Table 3 shows the results. Overall, patients with delayed care had a significantly greater mean CST at the first completed visit than at baseline (*P* = .0033). This trend was also seen in patients with nAMD and those with RVO who had delayed care (*P* < .0001 and *P* = .0467, respectively). In patients who completed the first scheduled visit, there was no significant difference in the mean CST compared with the baseline value.

**Table 3.** Mean CST (µm) Between Visits in Patients Who Completed a Scheduled Visit vs Those Who Had Delayed Care.

	Baseline Visit	First Completed Visit		Last Completed Visit	
Group	Mean CST ± SE	Mean CST ± SE	*P* Value^a^	Mean CST ± SE	*P* Value^b^
Entire cohort					
Overall	280 ± 3	284 ± 3	.1621	266 ± 3	<.0001^c^
Completed (n = 544)	278 ± 4	274 ± 4	.1218	267 ± 3	.0008^c^
Delayed (n = 379)	283 ± 5	298 ± 5	.0033^c^	264 ± 4	.0008^c^
nAMD					
Overall	258 ± 3	261 ± 4	.0232^c^	248 ± 3	.0039^c^
Completed (n = 357)	258 ± 3	253 ± 4	.0240^c^	247 ± 3	.0016^c^
Delayed (n = 240)	257 ± 4	273 ± 5	<.0001^c^	250 ± 4	.2394
DME					
Overall	326 ± 7	312 ± 9	.0232^c^	302 ± 7	.0024^c^
Completed (n = 66)	323 ± 14	304 ± 17	.0973	292 ± 13	.0168^c^
Delayed (n = 54)	330 ± 17	311 ± 19	.1141	304 ± 16	.0664
RVO					
Overall	319 ± 6	330 ± 7	.1066	296 ± 5	.0115^c^
Completed (n = 121)	313 ± 10	308 ± 11	.6709	305 ± 9	.4377
Delayed (n = 85)	329 ± 11	364 ± 13	.0467^c^	282 ± 11	.0049^c^

Abbreviations: CST, central subfield thickness; DME, diabetic macular edema; nAMD, neovascular age-related macular degeneration; RVO, retinal vein occlusion.

a Matched-pair test between baseline and first completed visit.

b Matched-pair test between baseline and last completed visit.

c Statistically significant.

One year later (at last completed visit), patients in the completed-care group and delayed-care group had a significantly lower mean CST than at the baseline visit (*P* = .0008 and *P* = .0008, respectively). In particular, the mean CST values were significantly lower at the last completed visit than at baseline in patients without delayed care with nAMD and those with DME (*P* = .0016 and *P* = .0168, respectively); this was also true for patients with delayed care and RVO (*P* = .0049). No group had significantly greater CST at 1 year.

### Etiology of Vision Loss

Table 4 shows the causes of vision loss of 15 or more ETDRS letters. A significantly greater proportion of patients who had a delay in care than patients who completed the first scheduled visit lost 15 or more ETDRS letters at the next completed visit (*P* < .0001). The main cause of vision loss in both groups was worsening retinal fluid. Other factors were neovascular causes (delayed-care group only) and atrophic causes.

**Table 4.** Causes of Vision Loss of ≥15 ETDRS Letters at Next Completed Visit^a^ and Last Completed Visit^b^.

Parameter	Number (%)		*P* Value^g^
	Completed	Delayed	
Next completed visit			<.0001^h^
Total^c^	15 (2)	54 (12)	
Cause of vision loss^d^			
Worsening retinal fluid	12 (80)	41 (76)	
Neovascular^e^	0	9 (17)	
Atrophic^f^	3 (20)	4 (7)	
Last completed visit			.1319
Total^c^	69 (10)	55 (12)	
Cause of vision loss^d^			
Worsening retinal fluid	32 (46)	25 (45)	
Neovascular^e^	12 (17)	7 (13)	
Atrophic^f^	25 (37)	23 (42)	

Abbreviation: ETDRS, Early Treatment Diabetic Retinopathy Study.

a Completed scheduled or next completed in case of the missed visit.

b One-year follow-up.

c Percentage of all patients with each diagnosis.

d Percentage of patients in each group who lost ≥15 EDTRS letters.

e Neovascular causes include tractional retinal detachment, vitreous hemorrhage, neovascular glaucoma, macular heme.

f Atrophic causes include atrophy, ischemia, fibrosis, scar.

g Chi-square test.

h Statistically significant.

At the 1-year follow-up, there was no statistically significant difference in the proportion of patients who lost 15 or more letters between the completed-care group and delayed-care group (*P* = .1319). The main cause of vision loss in both groups was worsening retinal fluid, followed closely atrophic causes. Neovascular causes were also a factor in visual loss in both groups.

### Number of Intravitreal Injections

Table 5 shows the number of intravitreal injections for 839 (72%) patients who initiated treatment on or before March 13, 2019, a year before the study period; the total number of injections received during the previous year (March 13, 2019, to March 15, 2020); and the number in the study year (March 16, 2020, to March 19, 2021). After correcting for multiple comparisons, there was no significant difference in the number of intravitreal injections during the previous year between the group with a delay in care and the group with no delay in care (*P* = .0491). In contrast, during the study year those with delayed care had significantly fewer injections than those with no delay in care overall (*P* < .0001); the difference remained significant when stratified by diagnosis.

**Table 5.** Comparison of the Number of Intravitreal Injections Received in the Prior Year and Study Year Between Patients Who Completed and Patients Who Missed the Scheduled Visit.^a^

Group	Mean Injections (n) ± SE		*P* Value^b^
	Completed	Delayed	
Overall cohort			
Prior year	7.0 ± .1	6.6 ± .2	.0491
Study year	6.4 ± .1	5.1 ± .2	<.0001^c^
nAMD			
Prior year	7.2 ± .1	7.0 ± .2	.2112
Study year	6.6 ± .1	5.4 ± .2	<.0001^c^
DME			
Prior year	5.8 ± .4	5.6 ± .4	.7274
Study year	5.3 ± .3	3.8 ± .4	.0032^c^
RVO			
Prior year	6.8 ± .3	6.2 ± .3	.1050
Study year	6.1 ± .3	5.2 ± .3	.0241^c^

Abbreviations: DME, diabetic macular edema; nAMD, neovascular age-related macular degeneration; RVO, retinal vein occlusion.

a Patients who initiated treatment during the pre-COVID year were excluded (pre-COVID year, March 13, 2019–March 15, 2020; COVID year, March 16, 2020–March 19, 2021).

b Student *t* test; Bonferroni adjustment performed for multiple comparisons.

c Statistically significant.

## Conclusions

The findings in this study provide a new understanding in the context of previously reported studies of the varied effects of interruptions in care on visual outcomes in patients with nAMD, DME, or RVO who require intravitreal injections. We found that short delays in care resulted in vision loss in these patients, which is consistent with findings reported in our previous study,^11^ and this highlights the importance of intravitreal injections in maintaining vision. We further examined the long-term VA outcomes and analyzed the difference in VA between baseline and follow-up values in each group.

Although patients with nAMD or RVO who missed a scheduled visit had short-term vision loss and patients with DME who missed a visit had less vision gain than those with no delay in care, there was no significant difference in VA 1 year later compared with the baseline visit. After adjusting for age, sex, and baseline VA, there was no significant difference in the change in VA at 1 year between patients who had a delay in care and patients who did not. In general, however, patients with nAMD lost vision at 1 year, whereas patients with DME or RVO maintained VA compared with baseline values.

Patients with nAMD who had a brief lapse in care experienced short-term vision loss; this loss was also observed after 1 year despite normalization of the mean CST. A retrospective study of 93 eyes of 77 patients with nAMD who were lost to follow-up (for 6 months or more)^13^ also found persistent declines in VA and normalization of central foveal thickness (CFT) 12 months following the return after a loss to follow-up. However, the study did not compare the visual outcomes with a control group of patients who were not lost to follow-up, making it difficult to assess whether the decline in VA was a result of the delay in care or a progression of chronic disease and atrophy. In the current study, patients with nAMD who did not have interrupted care also had vision loss at 1 year. Although intravitreal injections decrease vascular leakage and fluid burden within the macula, they do not control chronic disease progression, in particular in patients with nAMD.^14,15^

Patients with DME and timely care had vision gain that was maintained at 1 year, while patients who had a short delay in care had no statistically significant difference in vision outcomes or the mean CST over the short-term and 1 year after their return to follow-up. In a retrospective study of 90 eyes with DME that were lost to follow-up for 6 months or more,^16^ there was a modest decline in VA and increased CFT at the return visit but no difference in VA or CFT at the 3-month, 6-month, and 12-month visits. The vision loss observed immediately after return could be a result of the longer period of delayed care and/or the larger sample compared with that in the current study, although there was no control group to assess vision outcomes in patients with DME who were not lost to follow-up.

In another study of 82 patients with DME and a treatment lapse of more than 3 months and 82 control patients with DME,^17^ there was no difference in VA or CFT between the 2 groups after the lapse in treatment, although no comparison was made between return visits and baseline visits. The findings in this study further contribute to the existing literature and show that regular intravitreal injections are critical for promoting and maintaining vision improvements over the long-term in patients with DME.

Last, patients with RVO who had a delay in care regained vision lost over the short term; this is in contrast to the long-term vision loss in patients with nAMD. Patients with RVO who had delayed care also had a thinner mean CST at 1 year, while those who did not have a delay in care maintained both vision and the mean CST over the short term and long term. Overall, these findings are consistent with those reported in a study of 5-year VA outcomes in patients requiring intravitreal injections.^18^ That study found a gradual decline in VA in patients with nAMD and a gain in or maintenance of VA in patients with DME or RVO, even though the patients with nAMD received more injections on average.

Although VEGF is an important driver in the progression of all 3 disease states, there are important differences in the pathogenesis between nAMD, DME, and RVO that might explain the visual outcomes in patients receiving injections.^19^ In nAMD, oxidative stress within the RPE and outer retina as well as compromise of Bruch membrane are primary insults that ultimately drive the development of choroidal neovascularization and subretinal fibrosis. In DME, hyperglycemia results in damage to pericyte and endothelial cells of retinal vessels, resulting in progressive capillary dropout. With RVO, increased resistance in the retinal circulation drives increased VEGF levels as well as leukostasis. In both DME and RVO, macular ischemia can affect vision. Usually, interrupting the positive feedback loop induced by retinal nonperfusion can suppress macular edema and slow disease progression. Development of macular geographic atrophy also leads to vision loss in nAMD, which does not appear to be affected by anti-VEGF agents.

In this current study, we further found that in the short term, patients lost 15 or more ETDRS letters, mostly as a result of worsening retinal fluid. This was most pronounced in patients who had delayed care. In comparison, at 1 year there was no difference in the proportion of patients who lost 15 or more letters between those who completed the first scheduled visit and those who missed it. At this time, more patients lost vision as a result of chronic disease progression (atrophy, fibrosis, and ischemia). These findings are reflected in the mean CST trends in which patients with delayed care had a greater mean CST short term and both groups had a thinner mean CST at 1 year than at the baseline visit. Given that most patients were not treatment naïve at the initiation of the current study, these findings support previous reports of the causes of vision loss in patients requiring long-term treatment.^14,18,20,21^

The strengths of this study include a large sample and the use of a real-life cohort of retina patients seen at a multispecialty ophthalmology outpatient center and their associated imaging findings. Limitations of this study include its retrospective design and lack of information on disease and treatment durations. Although we examined the overall number of injections received in the prior year and study year and found a greater drop in the number of injections in the delayed-care group, this study lacks more granular information about subsequent visits, treatment intervals between visits, and whether the additional decrease in the number of injections (0.5) was a result of treatment futility or continued nonadherence. Also, by including only patients with a completed follow-up visit, we cannot report on the visual outcomes of patients who had much longer delays in care or were completely lost to follow-up.

In conclusion, we found that interruptions in care for patients with nAMD, DME, or RVO resulted in short-term VA loss, mostly as a result of increased retinal fluid. This highlights the importance of regular monitoring and treatment to maintain VA. However, there does not appear to be long-term differences in vision outcomes after treatment resumption in patients with short interruptions in care. After 1 year, patients with DME and without delayed care maintained vision gains that were not observed in patients with delayed care, while patients with RVO and delayed care regained vision that was lost and nAMD patients in both the on-time care and delayed-care groups experienced vision loss that possibly reflected chronic disease progression.

## Supplemental Material

sj-docx-1-vrd-10.1177_24741264221136637 – Supplemental material for Visual Acuity in Patients Requiring Intravitreal Injections: Short-Term and Long-Term Effects of Delay in Care Click here for additional data file. Supplemental material, sj-docx-1-vrd-10.1177_24741264221136637 for Visual Acuity in Patients Requiring Intravitreal Injections: Short-Term and Long-Term Effects of Delay in Care by Weilin Song, Emese Kanyo, Riley Bastian, Rishi P. Singh and Aleksandra V. Rachitskaya in Journal of VitreoRetinal Diseases

sj-jpg-2-vrd-10.1177_24741264221136637 – Supplemental material for Visual Acuity in Patients Requiring Intravitreal Injections: Short-Term and Long-Term Effects of Delay in Care Click here for additional data file. Supplemental material, sj-jpg-2-vrd-10.1177_24741264221136637 for Visual Acuity in Patients Requiring Intravitreal Injections: Short-Term and Long-Term Effects of Delay in Care by Weilin Song, Emese Kanyo, Riley Bastian, Rishi P. Singh and Aleksandra V. Rachitskaya in Journal of VitreoRetinal Diseases

sj-jpg-3-vrd-10.1177_24741264221136637 – Supplemental material for Visual Acuity in Patients Requiring Intravitreal Injections: Short-Term and Long-Term Effects of Delay in Care Click here for additional data file. Supplemental material, sj-jpg-3-vrd-10.1177_24741264221136637 for Visual Acuity in Patients Requiring Intravitreal Injections: Short-Term and Long-Term Effects of Delay in Care by Weilin Song, Emese Kanyo, Riley Bastian, Rishi P. Singh and Aleksandra V. Rachitskaya in Journal of VitreoRetinal Diseases

## References

[1] Moja L Lucenteforte E Kwag KH , et al. Systemic safety of bevacizumab versus ranibizumab for neovascular age-related macular degeneration. Cochrane Database Syst Rev. 2014;9:CD011230. doi:10.1002/14651858.CD011230.pub2 25220133 PMC4262120

[2] Sarwar S Clearfield E Soliman MK , et al. Aflibercept for neovascular age-related macular degeneration. Cochrane Database Syst Rev. 2016;2:CD011346. doi:10.1002/14651858.CD011346.pub2 26857947 PMC5030844

[3] Bahrami B Hong T Schlub TE Chang AA. Aflibercept for persistent diabetic macular edema: forty-eight-week outcomes. Retina. 2019;39(1):61-68. doi:10.1097/IAE.0000000000002253 30015767

[4] Mitchell P Bandello F Schmidt-Erfurth U , et al. The RESTORE study: ranibizumab monotherapy or combined with laser versus laser monotherapy for diabetic macular edema. Ophthalmology. 2011;118(4):615-625. doi:10.1016/j.ophtha.2011.01.031 21459215

[5] Nguyen QD Shah SM Khwaja AA , et al. Two-year outcomes of the ranibizumab for edema of the mAcula in diabetes (READ-2) study. Ophthalmology. 2010;117(11):2146-2151. doi:10.1016/j.ophtha.2010.08.016 20855114

[6] Clark WL Boyer DS Heier JS , et al. Intravitreal aflibercept for macular edema following branch retinal vein occlusion: 52-week results of the VIBRANT study. Ophthalmology. 2016;123(2):330-336. doi:10.1016/j.ophtha.2015.09.035 26522708

[7] Narayanan R Panchal B Das T , et al. A randomised, double-masked, controlled study of the efficacy and safety of intravitreal bevacizumab versus ranibizumab in the treatment of macular oedema due to branch retinal vein occlusion: MARVEL Report No. 1. Br J Ophthalmol. 2015;99(7):954-959. doi:10.1136/bjophthalmol-2014-306543 25631483

[8] Ehlken C Helms M Böhringer D Agostini HT Stahl A. Association of treatment adherence with real-life VA outcomes in AMD, DME, and BRVO patients. Clin Ophthalmol. 2017;12:13-20. doi:10.2147/OPTH.S151611 29339917 PMC5745150

[9] Weiss M Sim DA Herold T , et al. Compliance and adherence of patients with diabetic macular edema to intravitreal anti–vascular endothelial growth factor therapy in daily practice. Retina. 2018;38(12):2293-2300. doi:10.1097/IAE.0000000000001892 29068914

[10] Greenlee TE Wang VY Kang H , et al. Consequences of lapses in treatment with vascular endothelial growth factor inhibitors in neovascular age-related macular degeneration in routine clinical practice. Retina. 2021;41(3):581-587. doi:10.1097/IAE.0000000000002888 32658164

[11] Song W Singh RP Rachitskaya AV. The effect of delay in care among patients requiring intravitreal injections. Ophthalmol Retina. 2021;5(10):975-980. doi:10.1016/j.oret.2020.12.020 33395587

[12] Gregori NZ Feuer W Rosenfeld PJ. Novel method for analyzing snellen visual acuity measurements. Retina. 2010;30(7):1046-1050. doi:10.1097/IAE.0b013e3181d87e04 20559157

[13] Soares RR Mellen P Garrigan H , et al. Outcomes of eyes lost to follow-up with neovascular age-related macular degeneration receiving intravitreal anti-vascular endothelial growth factor. Ophthalmol Retina. 2020;4(2):134-140. doi:10.1016/j.oret.2019.07.010 31540854

[14] Rofagha S Bhisitkul RB Boyer DS Sadda SR Zhang K ; SEVEN-UP Study Group. Seven-year outcomes in ranibizumab-treated patients in ANCHOR, MARINA, and HORIZON: a multicenter cohort study (SEVEN-UP). Ophthalmology. 2013;120(11):2292-2299. doi:10.1016/j.ophtha.2013.03.046 23642856

[15] Campochiaro PA Sophie R Pearlman J , et al. Long-term outcomes in patients with retinal vein occlusion treated with ranibizumab: the RETAIN study. Ophthalmology. 2014;121(1):209-219. doi:10.1016/j.ophtha.2013.08.038 24112944

[16] Matsunaga DR Salabati M Obeid A , et al. Outcomes of eyes with diabetic macular edema that are lost to follow-up after anti–vascular endothelial growth factor therapy. Am J Ophthalmol. 2022;233:1-7. doi:10.1016/j.ajo.2021.06.028 34283979

[17] Yalamanchili SP Maatouk CM Enwere DU , et al. The short-term effect of a single lapse in anti–vascular endothelial growth factor treatment for diabetic macular edema within routine clinical practice. Am J Ophthalmol. 2020;219:215-221. doi:10.1016/j.ajo.2020.06.040 32640254 PMC7335491

[18] Wecker T Ehlken C Bühler A , et al. Five-year visual acuity outcomes and injection patterns in patients with pro-re-nata treatments for AMD, DME, RVO and myopic CNV. Br J Ophthalmol. 2017;101(3):353-359. doi:10.1136/bjophthalmol-2016-308668 27215744 PMC5339568

[19] Campochiaro PA. Retinal and choroidal vascular diseases: past, present, and future: the 2021 proctor lecture. Invest Ophthalmol Vis Sci. 2021;62(14):26. doi:10.1167/iovs.62.14.26 PMC8637787 34817536

[20] Iijima H. Mechanisms of vision loss in eyes with macular edema associated with retinal vein occlusion. Jpn J Ophthalmol. 2018;62(3):265-273. doi:10.1007/s10384-018-0586-5 29572577

[21] Bhisitkul RB Mendes TS Rofagha S , et al. Macular atrophy progression and 7-year vision outcomes in subjects from the ANCHOR, MARINA, and HORIZON studies: the SEVEN-UP study. Am J Ophthalmol. 2015;159(5):915-924.e2. doi:10.1016/j.ajo.2015.01.032 25640411

