# Supplementary material for: Visual Acuity in Patients Requiring Intravitreal Injections: Short-Term and Long-Term Effects of Delay in Care
Source: J Vitreoretin Dis. 2022 Dec 16;7(1):20–6. doi: 10.1177/24741264221136637 (PMC9954165; doi:10.1177/24741264221136637)
Supplement: sj-docx-1-vrd-10.1177_24741264221136637 – Supplemental material for Visual Acuity in Patients Requiring Intravitreal Injections: Short-Term and Long-Term Effects of Delay in Care [file sj-docx-1-vrd-10.1177_24741264221136637.docx]

**Supplemental Table 1. Change in VA between visits in patients who completed a scheduled visit vs those who had delayed care**

|  | Completed,  EDTRS letters(±SE) | Delayed,  EDTRS letters(±SE) | P-value^a^ |
| --- | --- | --- | --- |
| Change in VA from baseline to next completed visit (all) | 0.97 (±0.39) | -2.13 (±0.49) | ***<0.0001*** |
| nAMD | 0.11 (±0.39) | -1.34 (±0.50) | ***0.0217*** |
| DME and/or PDR | 4.18 (±1.33) | -1.42 (±1.61) | ***0.0147*** |
| RVO | 1.46 (±1.02) | -5.23 (±1.28) | ***<0.0001*** |
| Change in VA from baseline to last completed visit (all) | -0.68 (±0.51) | -1.57 (±0.68) | 0.3104 |
| nAMD | -1.76 (±0.60) | -2.44 (±0.78) | 0.4897 |
| DME and/or PDR | 4.68 (±1.86) | 1.72 (±2.24) | 0.3111 |
| RVO | -0.97 (±1.22) | -1.46 (±1.53) | 0.8046 |

a=student t-test. Bonferroni adjustment performed for multiple comparisons and significance level of p=0.025 used. VA = visual acuity. EDTRS = Early Treatment Diabetic Retinopathy Study. nAMD = neovascular age-related macular degeneration. DME = diabetic macular edema. PDR = proliferative diabetic retinopathy. RVO = retinal vein occlusion.
