# Supplementary figures and images for: Visual Acuity in Patients Requiring Intravitreal Injections: Short-Term and Long-Term Effects of Delay in Care
Source: J Vitreoretin Dis. 2022 Dec 16;7(1):20–6. doi: 10.1177/24741264221136637 (PMC9954165; doi:10.1177/24741264221136637)

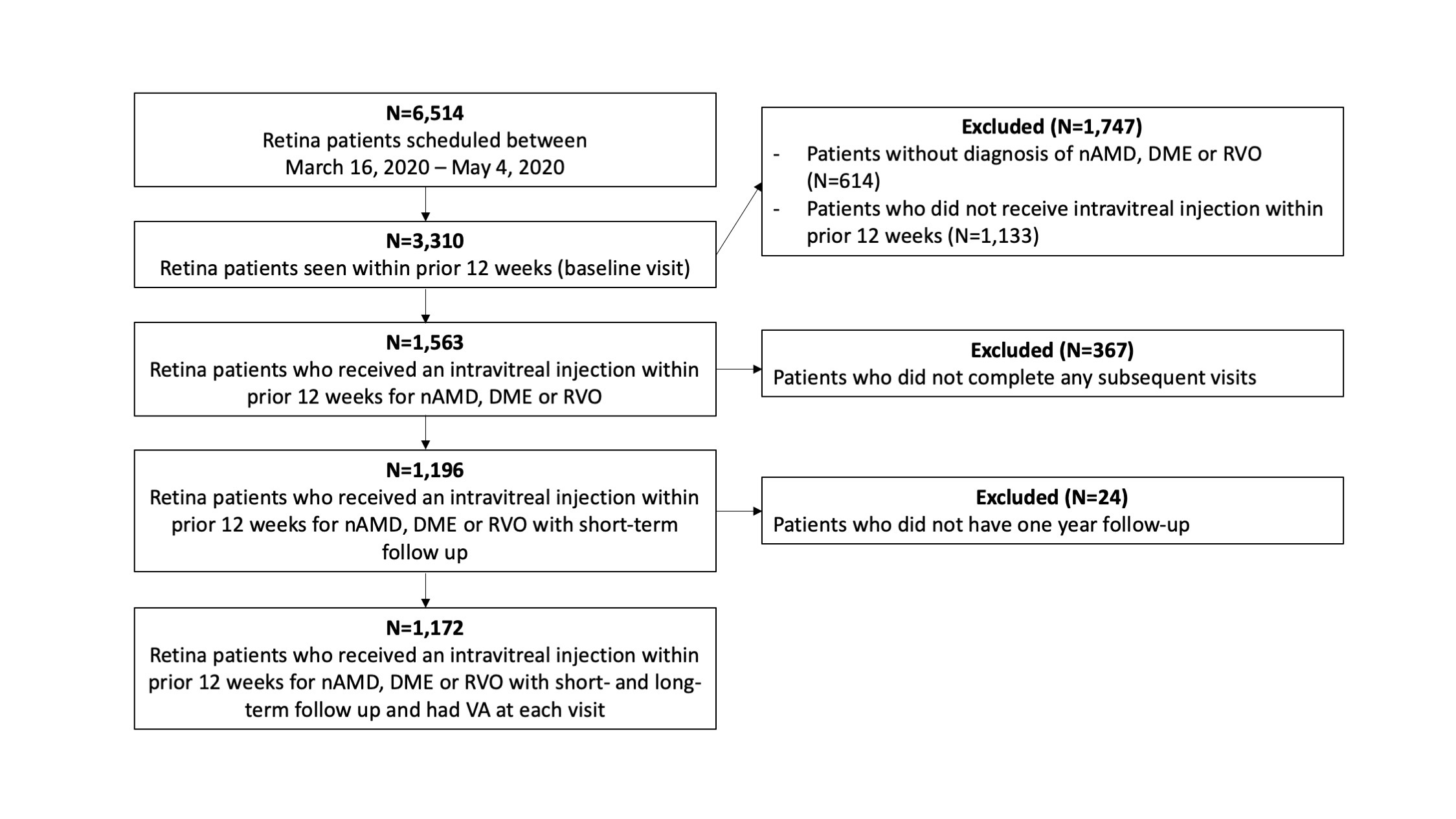

Supplement: sj-jpg-2-vrd-10.1177_24741264221136637 – Supplemental material for Visual Acuity in Patients Requiring Intravitreal Injections: Short-Term and Long-Term Effects of Delay in Care [file sj-jpg-2-vrd-10.1177_24741264221136637.jpg]

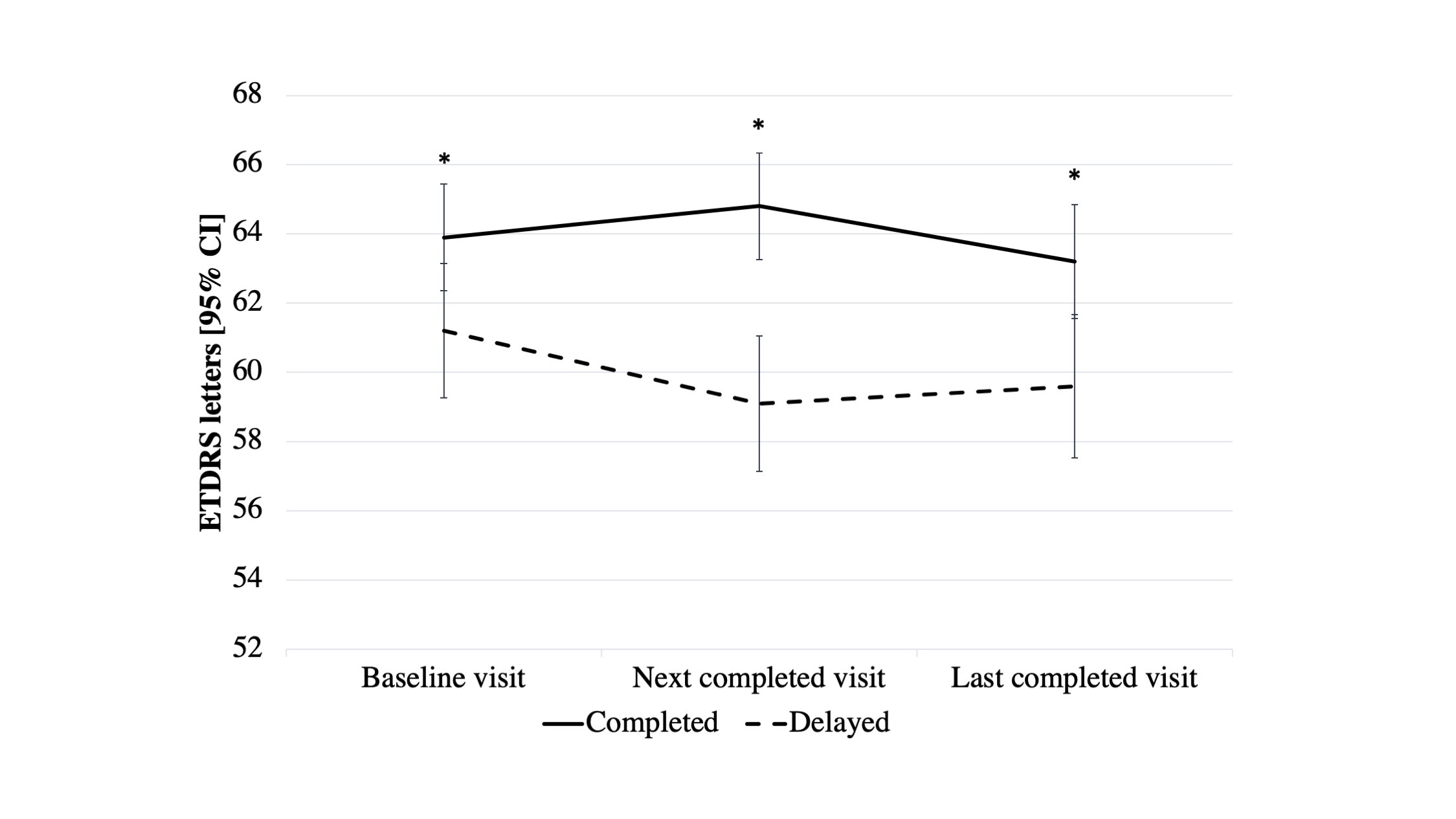

Supplement: sj-jpg-3-vrd-10.1177_24741264221136637 – Supplemental material for Visual Acuity in Patients Requiring Intravitreal Injections: Short-Term and Long-Term Effects of Delay in Care [file sj-jpg-3-vrd-10.1177_24741264221136637.jpg]
